# iPSC-modelling reveals genetic associations and morphological alterations of oligodendrocytes in schizophrenia

**DOI:** 10.1038/s41398-025-03509-x

**Published:** 2025-08-16

**Authors:** Man-Hsin Chang, Jan Benedikt Waldeck, Marius Stephan, Nirmal Kannaiyan, Valéria de Almeida, Emanuel Boudriot, Temmuz Karali, Lukas Röll, Laura Fischer, Damianos Demetriou, Nadia Gabellini, Sabrina Galinski, Andrea Schmitt, Sergi Papiol, Daniel Keeser, Peter Falkai, Moritz J. Rossner, Florian J. Raabe

**Affiliations:** 1 Department of Psychiatry and Psychotherapy, LMU University Hospital, LMU Munich, 80336 Munich, Germany; 2 International Max Planck Research School for Translational Psychiatry (IMPRS-TP), 80804 Munich, Germany; 3 Systasy Bioscience GmbH, 81669 Munich, Germany; 4 Department of Psychiatry, University of Münster, 48149 Münster, Germany; 5 Center for Soft Nanoscience, University of Münster, 48149 Münster, Germany; 6 Max Planck Institute of Psychiatry, 80804 Munich, Germany; 7 NeuroImaging Core Unit Munich (NICUM), LMU University Hospital, LMU Munich, 80336 Munich, Germany; 8 German Center for Mental Health (DZPG), partner site Munich/Augsburg, Munich, Germany; 9 Laboratory of Neurosciences (LIM-27), Institute of Psychiatry, University of São Paulo (USP), São Paulo-SP, 05403-903 Brazil; 10 Institute of Psychiatric Phenomics and Genomics (IPPG), LMU University Hospital, LMU Munich, 80336 Munich, Germany; 11 Munich Center for Neurosciences (MCN), LMU Munich, 82152 Planegg-Martinsried, Germany

**Keywords:** Schizophrenia, Stem cells, Molecular neuroscience

## Abstract

There is strong evidence for a genetically driven neuronal contribution in schizophrenia (SCZ). Although imaging and postmortem studies also provide evidence for white matter alterations with implications of the oligodendroglial lineage in SCZ, it is unclear whether these disturbances are a secondary consequence of neuronal deficits or also, at least in parts, genetically driven and cell-autonomous. Using human induced pluripotent stem cells (hiPSCs) in combination with gene set enrichment analysis, we investigated the cellular impact of SCZ genetics on the oligodendroglial lineage. We performed unsupervised clustering analysis of hiPSC-differentiated neural cells including oligodendrocytes (iOLs) and their precursor cells (iOPCs) with corresponding human postmortem cell types from single-cell RNA sequencing (scRNAseq) data and conducted a comparative gene set enrichment analysis. Subsequently, we stratified individuals based on white matter alteration using diffusion tensor imaging (DTI) within a translational cohort (N = 112) and then explored the cellular effects of SCZ risk with hiPSC modelling in a subset of SCZ patients (N = 8) with disturbed white matter integrity and unaffected healthy controls (N = 7). hiPSC-iOPCs/iOLs expression profiles strongly correlated with human postmortem OPCs/OLs based on scRNAseq, and their transcriptional signatures were highly enriched in the genetic associations of SCZ. The cellular assessment of patient-derived iOPCs/iOLs revealed morphological alterations, including significantly increased branch length and elevated junction number in mature iOLs from SCZ. Moreover, transcriptomic profiling revealed a dysregulation in oligodendroglial cell signaling and proliferation. In sum, hiPSC-modelling shows an impact of SCZ genetics on dedicated features of the oligodendroglial lineage.

## Introduction

Schizophrenia (SCZ) is a severe psychiatric disorder with a prevalence of 0.4-0.7% [1], and characterized by positive (e.g., delusions and hallucinations), negative (e.g., anhedonia, lack of motivation and social withdrawal) and cognitive symptoms (e.g., learning and attention deficits) [2]. While current antipsychotics mainly improve positive symptoms effectively [3], negative and cognitive symptoms remain an unmet medical need and the pathogenesis of SCZ requires a better understanding.

A recent multicenter imaging study has revealed widespread white matter disturbances in SCZ and suggested, based on the configuration of the diffusion tensor imaging (DTI) parameters, that these alterations are most likely driven by impaired myelination [4]. Of note, myelination and white matter development take place during early postnatal time, maintain through adolescence and complete in young adulthood, a critical period of the development of the central nervous system, coincident with the onset of SCZ in the majority of patients [5, 6]. Furthermore, white matter impairments in SCZ patients are associated with declined cognitive performances, likely mediated by deficits in processing speed [7, 8]. Oligodendrocytes (OLs) and their precursor cells (OPCs) are critically involved in myelin sheath formation, promoting fast saltatory conduction of action potentials along the axon, and facilitating connectivity and thus effective information processing between various brain regions. Moreover, oligodendroglial cells also provide metabolic support to neuronal cells, building cell-cell contact to allow axon-glia signaling, and influencing neuronal excitability [9, 10].

Apart from neuronal cells, oligodendroglial lineage cells also participate in cognitive processes, including learning and memory consolidation [11, 12], and impairments of oligodendrocyte functions are linked to cognitive deficits in SCZ [13, 14]. Postmortem studies have provided robust evidence for oligodendroglial alterations in SCZ, with reduced number of OLs, disturbed expression of OL-related genes and proteins, and altered myelination [13, 14]. Moreover, postmortem analyses indicate morphological changes in oligodendroglial cells from SCZ patients [15–17].

SCZ is a complex polygenic disorder with a multifactorial etiology with heritability estimates of about 79% [18]. Of note, post-hoc analyses based on genome-wide association studies (GWAS) in SCZ revealed the highest gene enrichment in cortical projection neurons and interneurons [19, 20]. However, moderate association of SCZ risk loci with gene sets from the oligodendroglial lineage were found with stronger effects from human postmortem data sets compared to mouse-derived transcriptomic data [19]. Pathway analysis that focused on glial lineages identified several risk-associated single nucleotide polymorphisms (SNPs) which are connected to genes that are expressed in OPCs/OLs [21, 22]. Post-hoc pathway and gene enrichment analyses of GWAS data face technical challenges for potential false-negative findings; i.e., transcriptomic data for GWAS-driven post-hoc analysis are based on human postmortem samples, which may not align with the most relevant cellular state or developmental stage, and murine expression profiles may differ in expression of critical genes [19, 20].

To investigate the cell-autonomous impact of SCZ on the oligodendroglial lineage in a human-derived model of living cells, we applied human induced pluripotent stem cell (hiPSC) modelling. The advantages of hiPSCs include their human origin and capability to differentiate into various cell types of interest, and to overcome the inaccessibility of living brain tissues in humans [23]. In a previous study, we successfully established a rapid and effective protocol for induced OL (iOL) differentiation [24] based on previous works [25, 26]. Here, we used scRNAseq data of hiPSC-derived late-stage iOPC/iOLs from our previous work [24] to conduct cell lineage-specific MAGMA gene enrichment analysis. Moreover, we investigated in a translational cohort of patient representatives with white matter disturbances based on DTI whether iOLs derived from individuals with SCZ display abnormal morphological characteristics and altered transcriptomic profiles compared to those derived from unaffected healthy controls.

## Materials and methods

### Ethics approval and consent to participate

This study was approved by the local ethics committee of the Ludwig Maximilian University Munich (votes 17–13, 17–880, 18–716, and 20–528). The entire study was conducted according to the Declaration of Helsinki and in accordance with the relevant guidelines and regulations. Written informed consent was obtained from all participants before taking part in the study.

### Clustering analysis

Pseudo-bulked count data tables were generated for datasets of hiPSC-derived SON-directed (overexpression of *SOX10*, *OLIG2*, and *NKX6.2*) oligodendroglial cells retrieved from GEO (GSE179516) in our previous work [24] and human postmortem brain cells retrieved from GEO (GSE118257) [27] using Seurat v3 [28] without log transformation. hiPSC data genes were pre-filtered based on a differential expression analysis run on sctransform-normalized, unintegrated data using *PrepSCTFindMarkers*, and then *FindAllMarkers*. *PrepSCTFindMarkers* corrected counts for technical differences between samples. The *FindAllMarkers* was not run on the integrated count data due to the smoothing effect of integration procedures which leads to the underestimation of transcriptional variances. The data were evaluated using the Wilcoxon rank-sum test and the *p*-values were FDR adjusted with Bonferroni correction. Marker genes for downstream analysis were identified with a minimal log_2_(fold change) of 0.25 between the individual cluster and the background and a minimum abundance of 10% of expressing cells. This step reduced the noise level and focused the analysis on genes differentiating the cell populations of interest. The filtered, and pseudo-bulked datasets were merged without prior integration, since *DESeq2* expects raw count data and relies on a modelling approach to address technical variability [29]. Next, gene expression variability was calculated using the R package *DESeq2* [29] for variance stabilization, and *removeBatchEffect* was run to correct for technical differences between samples. For downstream analysis, the 2000 most variable genes were selected. A principal component analysis (PCA) was performed to find clusters of samples, and Manhattan distances between samples and features were computed and used for hierarchical clustering and plotted as heatmaps.

### MAGMA gene enrichment analysis

MAGMA (Multi-marker Analysis of GenoMic Annotation, version 1.09) [30] was used to carry out formal enrichment analyses using the summary statistics of schizophrenia [20], bipolar disorder [31], and autism spectrum disorder [32] GWAS. Previously published single-cell RNA sequencing (scRNAseq) data from the SON-directed differentiation [24] were used to generate different gene sets (see Table S1) that characterize various cell and differentiation stages within the previously established protocol of directed oligodendroglial differentiation [24]: iPSC-derived neural precursor cells (iNPC), SON-induced OPCs (iOPC), SON-induced oligodendrocytes in different maturation stages iOL1 and iOL2 (more mature cluster), and iPSC-derived neurons (iNeuron) that escaped the directed oligodendroglial differentiation. After SNP-to-gene annotation, gene-level analyses (±10 kb) were carried out using the “SNPwise-mean” model. Gene-based *p*-values were obtained by combining the SNP *p*-values in a gene (±10 kb) into a gene test statistic (mean χ^2^) corrected for linkage disequilibrium between SNPs. MAGMA competitive gene-set analysis tested, within a regression framework, if the gene-sets in differentiating stages have a stronger association with the target phenotype than randomly selected gene-sets with similar characteristics. For gene-set competitive testing, linear regression analyses were conditioned to gene size, log(gene size), gene density, log(gene density), inverse mac and log(inverse mac).

### Magnetic resonance imaging (MRI) within clinical cohort

The clinical basis for this translational study were 58 patients with SCZ and 54 healthy controls (Ctrl) out of the *Multimodal Imaging in Chronic Schizophrenia Study* (MIMICSS), a pilot study of the *Munich Clinical Deep Phenotyping Study* [33], with processed DTI data out of a protocol of multimodal magnetic resonance imaging (mMRI). For detailed description of the MIMICSS cohort, please refer to the supplements of [33].

#### MRI acquisition

All MRI examinations were performed using a 3.0 T MR scanner (Siemens MAGNETOM Prisma, Siemens Healthineers AG, Erlangen, Germany) with a 32-channel head coil. T1-weighted scans were acquired using a magnetization-prepared rapid gradient echo (MP-RAGE) sequence with the following parameters: isotropic voxel size of 0.8 mm^3^, 208 slices, a repetition time (TR) of 2500 ms, an echo time (TE) of 2.22 ms, a flip angle of 8°, and a field of view (FoV) of 256 mm. DTI was performed with 95 non-collinear diffusion-encoding directions using a monopolar diffusion scheme. The DTI parameters were as follows: TR of 5500 ms, TE of 99.20 ms, FoV of 224 mm, voxel size of 1.6 mm^3^, slice thickness of 1.6 mm, and a multi-band acceleration factor of 3. Multiple diffusion weighting b-values were used (b = 0 s/mm^2^ and 2000 s/mm^2^).

#### Preprocessing and tensor fitting

Diffusion-weighted images (DWIs) were preprocessed using the *FMRIB Software Library* (FSL, version 6.0.7.6) [34]. The preprocessing pipeline included eddy current and motion correction of the DWIs using the eddy correct tool. Brain extraction was performed using the *Brain Extraction Tool* (BET) to exclude non-brain tissues. Subsequently, the diffusion tensor model was fitted at each voxel using the *DTIFIT* command, which involved the following parameters: input diffusion data, output basename for the files, brain mask, gradient directions file, and b-values file. The outputs from this step included fractional anisotropy (FA), three eigenvalues (L1, L2, L3), and eigenvectors.

#### Tract-based spatial statistics (TBSS)

TBSS was utilized to perform voxelwise statistical analysis of the FA data following the standard FSL pipeline [35]. The TBSS analysis comprised several steps: (1) all FA images were aligned to a common space using the *tbss_1_preproc* script, (2) non-linear registration: the images were non-linearly registered to the *FMRIB58_FA* standard-space image using the *tbss_2_reg -T* command, (3) post-registration: a mean FA image was created and thinned to produce a mean FA skeleton representing the centers of all tracts common to the group using the *tbss_3_postreg -S* command, and (4) thresholding: the mean FA skeleton was thresholded at FA > 0.2 using the tbss_4_prestats 0.2 command.

#### Statistics

Voxelwise statistical analysis was performed using the randomize tool with 5000 permutations to test for significant differences across groups, with multiple comparisons corrected using threshold-free cluster enhancement (TFCE). FA values from the skeleton were extracted for each subject for further analysis using the *fslstats* command, and then evaluated for normality using Shapiro-Wilk tests and QQ-plots and assessed for homogeneity of variances with Levene’s test. Next, comparison between SCZ and Ctrl group was performed with t-test. The test results can be found in Table S2.

### hiPSC cohort

hiPSCs utilized in this study were from translational patient representatives out of a characterized hiPSC cohort [36] that is part of deep phenotyped clinical cohorts within the Munich Mental Health Biobank [33] located at the Department of Psychiatry and Psychotherapy, LMU University Hospital, LMU Munich, Germany (for selection procedure, see results below). Diagnosis of patients with SCZ was assessed according to the *International Statistical Classification of Diseases and Related Health Problems, 10*^*th*^ *revision (ICD-10)* in clinical settings. Guided by meta-analytical driven power calculations [37], we increased the number of included individual donors up to recommended 7/8 donors per group rather than analyzing multiple clones from very few donors to enhance the generalizability of our study.

These hiPSCs were created from peripheral blood mononuclear cells (PBMCs) following a previously established procedure [38]. Standard verification of hiPSCs involved confirming their pluripotency through immunocytochemistry (utilizing markers TRA1-60, NANOG, OCT4, and SOX2), assessing genomic integrity via digital karyotyping [39] (pipeline accessible at https://gitlab.mpcdf.mpg.de/luciat/cnv_detection.git, last accessed on June 28, 2021), and demonstrating successful differentiation into all three germ layers [40]. Additionally, the hiPSCs were screened for the absence of HIV, HCV, and CMV (by Synlab, Munich, Germany), and were confirmed to be free from mycoplasma infections (by Eurofins, Ebersberg, Germany). All lines were registered at the human pluripotent stem cell registry (hPSCreg®), register codes can be found in Table S2.

### hiPSC cultivation

hiPSCs were cultured in feeder-free iPS-Brew medium (Miltenyi Biotec, Bergisch Gladbach, Germany, 130-104-368) on vitronectin (Thermo Fisher Scientific, Waltham, MA, USA, A14700)-coated plate.

### Lentiviral infection and selection

hiPSCs with a concentration of 5 × 10^4^ cells/cm^2^ were seeded in iPSC-Brew medium supplemented with 1 μM ROCK inhibitor Y-27632 2HCl (Selleckchem, Houston, TX, USA, S1049). Lentiviral vectors containing doxycycline-inducible operator (tetO)-controlled SOX10-P2A-OLIG2-T2A-NKX6.2 (SON) plasmid and constitutively expressed reversed tetracycline transactivator (rtTA) followed by a puromycin selection cassette were used to infect hiPSCs. 48 hr after infection, cells were selected with 1 μg/ml puromycin (Thermo Fisher Scientific, A1113803).

### Neural induction and oligodendroglial differentiation

Neural induction and oligodendroglial differentiation were performed as previously described [24]. In short: iPSC-Brew medium was changed to mTeSR1 medium (StemCell, Vancouver, Canada, 85850) at least one passage before neural induction. hiPSCs were singularized using Accutase (Sigma-Aldrich, St. Louis, USA, A6964) and seeded with a concentration of 2 × 10^4^ cells/cm^2^ in mTeSR1 medium supplemented with 1x RevitaCell (Thermo Fisher Scientific, A2644501) on Matrigel (BD Bioscience, San Jose, CA, USA, 354277)-coated 12- and 24-well plates. After two days, mTeSR1 medium was changed to N2B27 medium, comprising DMEM/F-12 with GlutaMAX™ (Thermo Fisher Scientific, 31331028), 1x N2 (Thermo Fisher Scientific, 17502048), 1x B27 (Thermo Fisher Scientific, 12587010), 1x NEAA (Thermo Fisher Scientific, 11140035), 50 μM mercaptoethanol (Thermo Fisher Scientific, 21985023) and 25 μg/ml insulin (Sigma-Aldrich, I9278), supplemented with 10 μM SB431542 (StemCell, 72232), 1 μM LDN193189 (StemCell, 72147) and 0.1 μM retinoic acid (RA) (Sigma-Aldrich, R2625). Full media change was performed daily for 12 days, 1 ml/well and 0.5 ml/well for 12- and 24-well plates, respectively. The volume of media was doubled four days after neural induction. Eight days after neural induction, N2B27 medium supplemented with 0.1 μM RA and 1 μM SAG (Millipore, Burlington, MA, USA, 566660) was used instead.

Day 12 hiPSC-derived neural precursor cells (iNPCs) were singularized using Accutase and seeded with a concentration of 1.5 × 10^5^ cells/cm^2^ in N2B27 medium supplemented with 1x RevitaCell, 0.1 μM RA and 1 μM SAG on poly-L-ornithine (Sigma-Aldrich, P4957) and laminin (Sigma-Aldrich, L2020)-coated plates. Next day, N2B27 medium was changed to OL differentiation medium which is N2B27 medium supplemented with 10 ng/ml PDGF-AA (PeproTech, Waltham, MA, USA, 100-13 A), 10 ng/ml IGF1 (PeproTech, 100-11), 5 ng/ml HGF (PeproTech, 100-39), 10 ng/ml NT3 (Peprotech, AF-450-03), 0.1 ng/ml biotin (Sigma-Aldrich, B4639), 1 mM dbcAMP (Sigma-Aldrich, D0627), 60 ng/ml T3 (Sigma-Aldrich, T6397). Full media change was performed every other day. From day +0 to +6, the medium was supplemented with 1 μg/ml doxycycline (Clontech, Shiga, Japan, NC0424034) to induce the overexpression of the transcription factors SOX10, OLIG2, and NKX6.2 (SON) promoting the differentiation of iOLs. From day +4 to +6, cells were additionally selected for the SON-construct using 1 μg/ml puromycin. Differentiated iOLs obtained from one parallel differentiation were examined by immunocytochemistry (ICC) on Day +10.

### Immunocytochemistry

Cells seeded on glass coverslips were washed with PBS once and then fixed with 4% PFA for 10 min. After being washed with PBS three times, cells were permeabilized and blocked with 0.1% Triton-X (Sigma-Aldrich, T8787) and 5% goat serum (Thermo Fisher Scientific, 16210064) in PBS for 1 hr. To be noted, Triton-X was not used in all processes for O4 staining. Cells were incubated with primary antibodies in antibody solution (0.1% Triton-X and 1% goat serum in PBS) at 4 °C overnight. After three times of PBS wash, cells were incubated with secondary antibodies in antibody solution and protected from light for 1 hr. Cells were subsequently counterstained with DAPI (BD Bioscience, 564907) for 5 min and washed with PBS twice. Lastly, cells on coverslips were mounted to the microscope slides with a mounting medium. Cells were imaged using Axio Observer Z1 inverted fluorescence microscope (Zeiss, Oberkochen, Germany). Each PBS wash took 5 min. All steps were conducted at room temperature unless otherwise stated.

The following primary antibodies were used: mouse anti-O4 (1:200, R&D Systems, Minneapolis, MN, USA, MAB1326) and chicken anti-MBP (1:50, Millipore, AB9348). Secondary antibodies used: Alexa 555 goat anti-mouse IgM (1:500, Thermo Fisher Scientific, A21426) and Alexa Plus 488 goat anti-chicken (1:500, Thermo Fisher Scientific, A32931).

### Image analysis

To perform quantitative image analysis, we developed a customized morphology analysis pipeline (for the applied macro script see **Code Availability**) with the BioVoxxel toolbox [41] based on Fiji, a distribution of ImageJ [42].

#### Image processing

Raw images were first converted to 8-bit and split into each channel (DAPI and O4, DAPI and MBP). To correct the bleed-through of DAPI signals in MBP images, regions of interest (ROIs) in both channels were defined. First, the extended particle analyzer was applied to MBP images, and only ROIs with a solidity < 0.7 were retained. Next, ROIs of MBP images having less than 60% overlap with ROIs of DAPI images were kept using the binary feature extractor, rendering the MBP images free of DAPI bleed-through. The uneven illumination was then adjusted through the pseudo-flat field correction for MBP and O4 channels. Next, all images were smoothed using the median filter, followed by a background subtraction for O4 and DAPI images with the sliding paraboloid method. The contrast of DAPI and O4 images was enhanced subsequently. Different thresholding approaches were used after optimizing for each channel: the Huang2 method was used on DAPI and MBP images; the Huang method [43] was used on O4 images. Finally, several binary operations were adopted: Erode, Open, Dilate and Watershed commands were used for DAPI images; Erode and Open commands for O4 images.

#### Image analysis and statistics

After processing of images, the percentages of O4-/MBP-positive (O4^+^/MBP^+^) cells were quantified by dividing the numbers of O4^+^/MBP^+^ cells colocalizing with DAPI by total DAPI^+^ cells. The average cell size was determined by dividing the total area with a positive signal by the total cell number. For morphological analysis, *ridge detection* [44] and *skeletonize* [45] algorithms were used to quantify the branch length and junction number.

All data are shown in mean ± SD. The n numbers in image analysis indicate the total images examined in each group, unequal n numbers resulted from the NA signals produced in some analyses. All statistical analyses were performed in R (version 4.2.3). Data were first transformed to normal distribution by optimal methods, such as Yeo-Johnson, Box-Cox, orderNorm, sqrt(x + a), and asinh(x) [46], and then the normalized data were evaluated for normality using Shapiro-Wilk tests and QQ-plots. Extreme outliers and homogeneity of variances were assessed with Levene’s test, and homogeneity of covariances were assessed with Box’s M test. To analyze morphological differences, we applied mixed-effects ANOVA, a recommended and widely used statistical method for handling complex experimental designs involving repeated measures and hierarchical data structures [47]. Mixed-effects models are particularly suited for experiments where data are nested at multiple levels, as they account for both between-group differences (SCZ vs. Ctrl) and within-group variability (individual variation of the iPSC lines). This approach provided the sensitivity needed to detect significant group-level effects despite variability among individual lines.

For further subanalysis, independent t-tests were performed on group-level averages, and effect sizes were calculated using Cohen’s d [48]. Significance was defined as *, *p* < 0.05.

### Bulk RNA sequencing (RNAseq) and gene set enrichment analysis (GSEA)

5 × 10^5^ Day +10 iOLs were lysed in 350 µl Buffer RLT (Qiagen, Venlo, Netherlands, 74104) using QIAshredder homogenizer (Qiagen, 79656), and their RNAs were extracted by RNeasy Kits (Qiagen, 74104). cDNA synthesis was conducted using SMART-Seq® v4 Ultra® Low Input RNA Kit for Sequencing (Takara Bio, Kusatsu, Japan, 634893) and the library preparation was performed by Nextera XT DNA Library Preparation Kit (Illumina, San Diego, CA, USA, FC131-1096). The sequencing was run in NovaSeq 6000 Sequencer (Illumina).

The quality of raw reads was examined using *FastQC* (version 0.11.9), and mapped to Ensembl GRCh38 human genome by *STAR aligner* (version 2.7.10a) [49]. Next, the mapped reads were counted for corresponding genes using *featureCounts* (Version 2.0.1) [50]. The data were subsequently analyzed with R package *DESeq2* [29] to find differentially expressed genes with the threshold set for *p*-value < 0.05 and log_2_FoldChange >= 1.5 or log_2_FoldChange <= −1.5.

Given the highly correlated structure of the transcriptomic data, not only the conventional false discovery rate (FDR) correction but also an alternative *p*-value correction based on estimating the effective number of tests using the *simpleM* method [51] implemented in R package *poolr* was used. The gene expression matrix was further used for revealing relevant pathways by gene set enrichment analysis (GSEA) [52] based on the full transcriptome.

## Results

### hiPSC-derived iOPCs/iOLs cluster with human postmortem OPCs/OLs

**Fig. 1:**
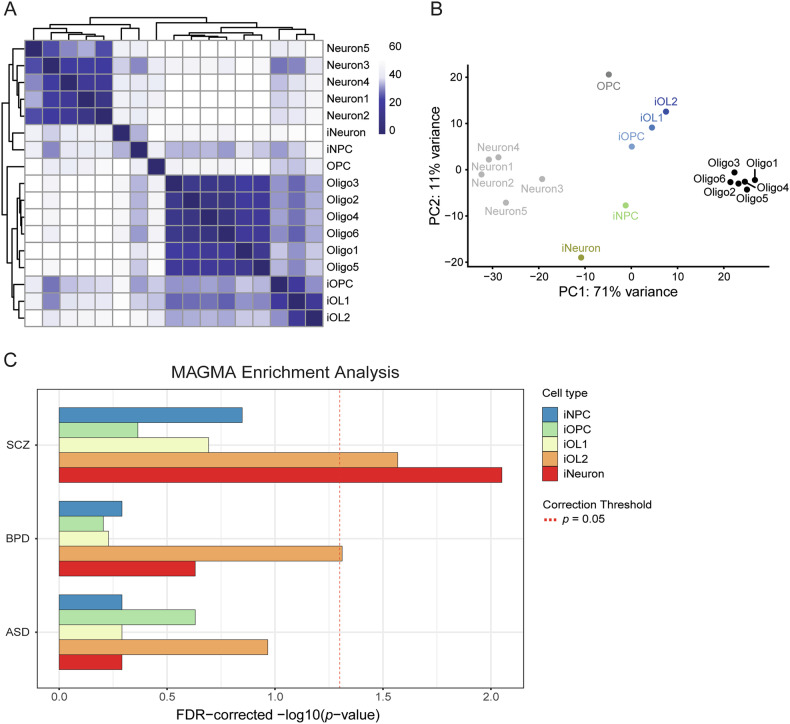
hiPSC-derived induced oligodendrocyte precursor cells (iOPCs) and induced oligodendrocyte (iOLs) cluster with human postmortem OPCs and OLs, and genes of mature iOLs are enriched in schizophrenia (SCZ) GWAS. **A** Comparison of the transcriptome of hiPSC-derived oligodendroglial cells from Raabe et al. [24] with human postmortem brain cells from Jäkel et al. [27]. Heatmap of Manhattan sample distances and dendrograms from unsupervised hierarchical clustering of scRNAseq data. **B** Clustering of scRNAseq data with dimensionality reduction by PCA. hiPSC-derived cells are colored, whereas reference postmortem samples are in black and grey. **C** MAGMA gene-set enrichment analysis of different cell and differentiation stages in different psychiatric disorders based on scRNAseq data from a directed oligodendroglial differentiation [24]. SCZ, schizophrenia; BPD, bipolar disorder; ASD, autism spectrum disorder; OPCs, human postmortem oligodendrocyte precursor cells; Oligo1-6, clusters of human postmortem oligodendrocytes; Neuron1-5, clusters of human postmortem neurons; iOPC, hiPSC-derived OPC; iOL1-2, hiPSC-derived oligodendrocytes; iNPC, hiPSC-derived neural precursor cell; iNeuron, hiPSC-derived neurons.

To prove the validity of hiPSC-derived late-stage iOPCs and iOLs generated by the previously described protocol [24], we investigated whether their transcriptional profiles cluster with that of human postmortem OPCs and OLs [27]. Unsupervised clustering analyses demonstrate that iOLs and iOPCs are related to human postmortem OLs and OPCs, while hiPSC-derived induced neuronal cells (iNeurons) and neural precursor cells (iNPCs) segregate completely from the oligodendroglial lineage and co-cluster with human postmortem neurons (Fig. 1A), indicating that these two groups of cells display similar expression across all genes. Similarly, dimensional reduction with PCA separated two major sub-clusters, iOLs/iOPCs with human postmortem OLs/OPCs and iNeurons/iNPCs with human postmortem neurons, along PC1 by 71% variance (Fig. 1B). Furthermore, the hierarchical cluster analysis of the 50 genes whose expression varied most across samples showed higher correlated gene expression profiles in iOLs and mature OL subgroups (Oligo1-6), and displayed highly upregulated expression levels of *PLP1*, *QKI*, *MBP* and *CNP*, which are the marker genes for maturating OLs (Supplementary Fig. 1). These results confirm the strong similarity of the transcriptional profiles between iOPCs/iOLs and human postmortem OPCs/OLs.

### MAGMA gene enrichment analysis indicates OL gene enrichment in SCZ

Most psychiatric disorders are characterized by a complex polygenic architecture, with hundreds to thousands common genetic variants contributing only with small effects to their etiology [53]. To investigate if gene expression profiles at different differentiation stages are associated with SCZ risk, we analyzed if cell-type specific gene sets, that we have obtained by in depth scRNAseq profiling of neural cells with a previously established oligodendroglial differentiation protocol [24], are enriched in genetic associations identified in SCZ GWAS [20]. Moreover, we included two more psychiatric disorders with high heritability estimates to our analysis: bipolar disorder (BPD) [31], and autism spectrum disorder (ASD) [32]. MAGMA gene enrichment analysis demonstrated that apart from neuronal genes, genes enriched in iOL2 were associated with SCZ (Fig. 1C, Table S1). Of note, iOL1 and iOL2 are two distinct iOL clusters, with iOL2 representing a cluster of more mature OLs [24]. Moreover, iOL2 genes were also associated with BPD risk, although with a less significant *p*-value.

### Identification of SCZ patients with white matter disturbances

Patient stratification was performed within the translational *Multimodal Imaging in Chronic Schizophrenia Study* (MIMICSS), the pilot study of the *Munich* *Clinical Deep Phenotyping* (CDP) study [33], based on DTI imaging.

**Fig. 2:**
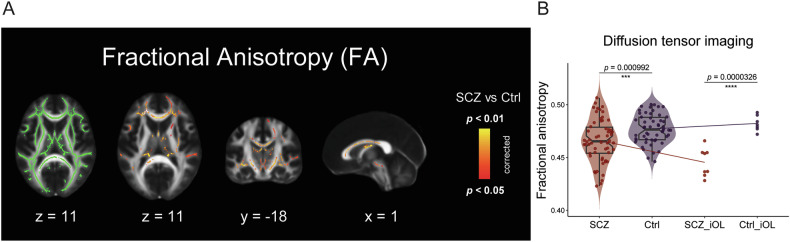
Diffusion tensor imaging (DTI) reveals decreased fractional anisotropy (FA) in SCZ and allows patient stratification. **(A)** Investigation of the FA of the whole brain white matter skeleton with tract-based spatial statistics (TBSS) based on DTI in patients with SCZ (N = 58) compared to unaffected healthy controls (Ctrl; N = 54). Investigated areas are displayed in green on the left, areas with significant lower FA values in SCZ compared to Ctrl are depicted in a red-yellow scale from the second to fourth brain images. X, y, and z indicate the brain map coordinates. **(B)** Violin plots illustrate quantification of whole brain FA based on TBSS in SCZ patients and Ctrl. FA values of the patient and Ctrl representatives that are further investigated with iOL are individually illustrated on the right. Data = Mean ± SD. T-test: ***, *p* < 0.001; ****, *p* < 0.0001.

TBSS of DTI revealed an altered integrity of whole skeleton white matter tracts in SCZ patients (N = 58) compared to unaffected healthy controls (Ctrl, N = 54; Fig. 2A, Table S2). In detail, within the clinical cohort, the FA of patients with SCZ was reduced compared to Ctrl (Fig. 2B**left**, SCZ = 0.466 ± 0.02 vs. Ctrl. 0.477 ± 0.014, *p* < 0.001), indicating white matter pathology in SCZ. Based on the FA stratification within the clinical cohort, we further selected seven SCZ patients with reduced FA and eight unaffected healthy controls of whose hiPSCs were available from a characterized cohort of mental illness [36] (Table S3). Within this stratified translational hiPSC cohort, stronger discrepancy in FA values was shown between the investigated groups (Fig. 2B**right**, SCZ = 0.445 ± 0.013 vs. Ctrl = 0.482 ± 0.007, *p* < 0.0001).

### Oligodendroglial differentiation

**Fig. 3:**
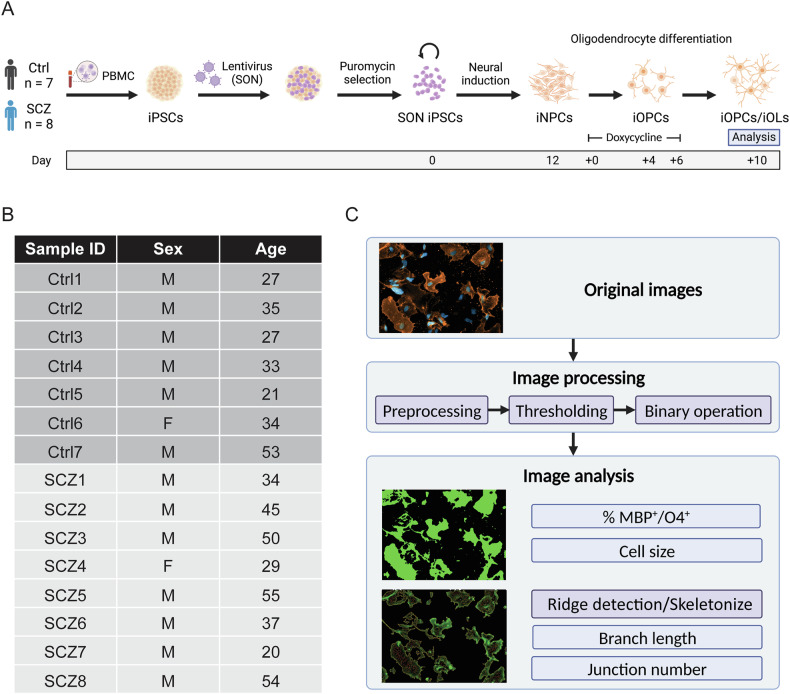
Schematic overview of the experimental study design. **A** Overview scheme of directed iOL differentiation [24] from human peripheral blood mononuclear cell (PBMC)-derived iPSC-lines with initial neural induction to induced neural precursor cells (iNPC) and subsequent induced overexpression of the transcription factors *SOX10*, *OLIG2*, and *NKX6.2* (SON) to generate O4^+^ induced oligodendrocyte precursor cells (iOPCs) and MBP^+^ induced oligodendrocytes (iOL). **B** Basic cohort information. **C** Overview workflow of image analysis with Fiji. M, male; F, female; MBP, myelin basic protein.

To investigate if the revealed genetic association of SCZ with OLs could contribute to cellular oligodendroglial effects, we examined iOPCs/iOLs derived from the stratified SCZ patients with presumed white matter disturbances compared to Ctrl (Fig. 3A, and B; Table S3). After neural induction and subsequently induced oligodendroglial differentiation with overexpression of the OL lineage transcription factors *SOX10*, *OLIG2* and *NKX6.2* (SON), we examined the number and the morphology of O4^+^ late-stage iOPCs/premyelinating iOLs and MBP^+^ mature iOLs using an automated image analysis pipeline (Fig. 3C). All cell lines derived from Ctrl and SCZ subjects were successfully differentiated into the oligodendroglial lineage, validated by the positive staining of O4 as a marker for OPCs (Supplementary Fig. 2). Maturation to iOLs was validated by the positive staining of myelin basic protein (MBP) for almost all cell lines from Ctrl and SCZ. However, one SCZ cell line (SCZ7) failed to differentiate into iOLs and was excluded from the morphological analyses of iOL (Supplementary Fig. 3).

### No morphological changes of O4^+^ iOPCs in SCZ

**Fig. 4:**
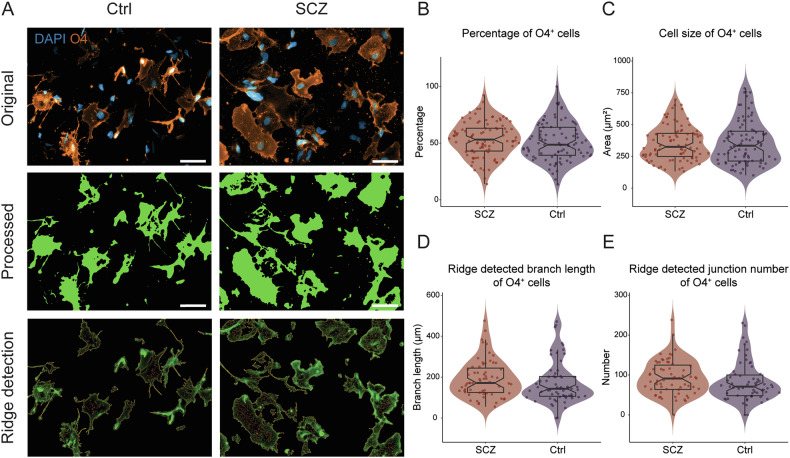
Image analysis reveals neither alteration of total amount nor morphological changes in O4^+^ oligodendrocyte precursor cells in SCZ. **A** Representative original (top), processed (middle), and *ridge detection* (bottom) images of O4^+^ iOPCs from SCZ patients and Ctrl. Boxplots illustrate the **(B)** percentage of O4^+^ cells (Ctrl, N = 7, n = 13 fields of view / cell line, vs. SCZ, N = 8, n = 10–13 fields of view / cell line) and the quantification of **(C)** average cell size (Ctrl, N = 7, n = 12–13 fields of view / cell line vs. SCZ, N = 8, n = 10–13 fields of view / cell line), as well as the morphological quantification with *ridge detection* of **(D)** average branch length and **(E)** average junction number (Ctrl, N = 7, n = 10 fields of view / cell line, vs. SCZ, N = 8, n = 8–10 fields of view / cell line). Data are based on biological independent hiPSC lines from 8 patients with SCZ and 7 Ctrl. Scale bar indicates 50 μm, Data = Mean ± SD. Mixed-effects ANOVA with group identity (SCZ-Ctrl) as the between-group factor.

To investigate whether O4^+^ iOPCs display numerical or morphological alterations between the groups, images of O4^+^ iOPCs were processed by an automated imaging pipeline (Fig. 3C, Fig. 4A). However, neither the number (Fig. 4B) nor the average size (Fig. 4C) of O4^+^ iOPCs was different between Ctrl and SCZ. Moreover, in the more detailed morphological quantification, we did not find significant differences in the average branch length (Fig. 4D) and the average junction number (Fig. 4E) using the image processing method *ridge detection* [44] nor by applying *skeletonize* [45] as quantification method of fine structures of the iOL morphology (Supplementary Fig. 4A-C, Supplementary Fig. 5A and B; see Table S2 for statistical parameters). Thus, by applying quantitative image analysis methods, we could not reveal significant numerical or morphological alterations in O4^+^ iOPCs from SCZ patients compared to Ctrl.

### Quantitative image analysis shows morphological alterations in MBP^+^ iOLs from SCZ patients

**Fig. 5:**
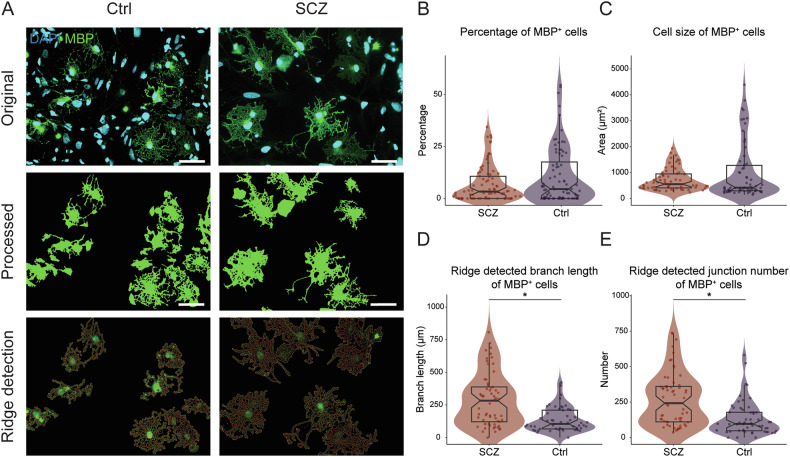
Image analysis reveals morphological alterations of MBP^+^ oligodendrocytes in SCZ. **(A)** Representative original (top), processed (middle), and *ridge detection* (bottom) images of MBP^+^ iOLs from SCZ patients and Ctrl. Boxplots illustrate the **(B)** percentage of MBP^+^ iOLs (Ctrl, N = 7, n = 9–11 fields of view / cell line, vs. SCZ, N = 7, n = 9–11 fields of view / cell line) and the **(C)** quantification of average cell size (Ctrl, N = 7, n = 8–11 fields of view / cell line, vs. SCZ, N = 7, n = 8–11 fields of view / cell line), and the morphological quantification with *ridge detection* of **(D)** average branch length and **(E)** average junction number (Ctrl, N = 7, n = 8–10 fields of view / cell line, vs. SCZ, N = 7, n = 8–10 fields of view / cell line). Data are based on biological independent hiPSC lines from 7 patients with SCZ and 7 Ctrl. Scale bar indicates 50 μm, Data = Mean ± SD. Mixed-effects ANOVA with group identity (SCZ-Ctrl) as the between-group factor: *, *p* < 0.05.

To investigate whether maturating iOLs display numerical or morphological alteration, we applied a customized automated quantification pipeline to the images from MBP^+^ mature iOLs (Fig. 3C; Fig. 5A). First, neither the average amount (Fig. 5B) nor the average cell size of MBP^+^ iOLs (Fig. 5C) was significantly altered in SCZ cell lines. To further examine if there are morphological changes in processes and fine structures of iOLs from SCZ patients, we performed quantification analysis of the cellular morphology using the *ridge detection* approach [45]. We found significantly higher average branch length (Fig. 5D, 0.676 ± 0.151 vs. 0.624 ± 0.164, *p* = 0.011) and increased average junction number (Fig. 5E, 0.511 ± 0.162 vs. 0.409 ± 0.225, *p* = 0.038) in iOLs from SCZ patients compared to Ctrl (see Table S2 for statistical parameters). For technical validation, we performed the morphological quantification analysis with the *skeletonize* approach [44] as an alternative imaging analysis method and could confirm, although not completely equivalent, the detected morphological alterations with higher branch length (Supplementary Fig. 4D and E). Although an additional t-test comparison of group-level averages without accounting for the applied repeated measurements per individual did not reach significance, Cohen’s d revealed a small effect size for branch length (Supplementary Fig. 5C, Table S2, Cohen’s d = 0.37) and a medium effect size for junction number (Supplementary Fig. 5D, Table S2, Cohen’s d = 0.52). Notably, while variation was observed across the hiPSC lines, the significant effects in MBP^+^ iOLs were not driven by a few extreme cell lines but were instead representative of consistent group-level trends among hiPSC lines from patients with schizophrenia (Supplementary Fig. 5). In sum, our quantification analyses of MBP^+^ iOLs suggest that SCZ exhibits altered cell morphology in maturating iOLs.

### Transcriptomic analyses reveal disturbed pathways involved in cell signaling and replication in iOLs from SCZ patients

**Fig. 6:**
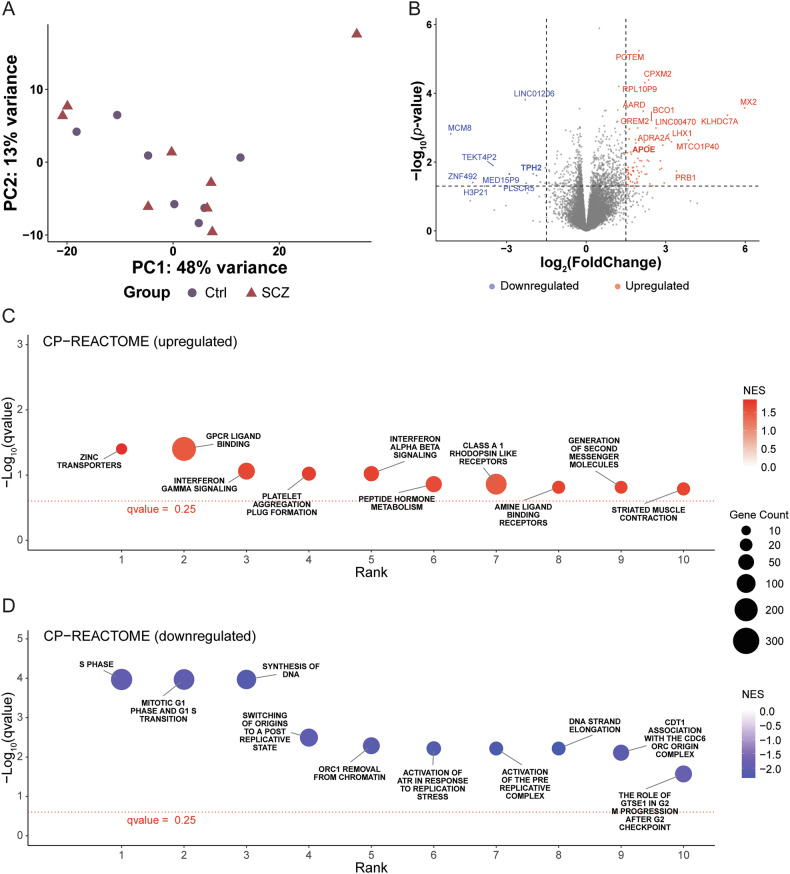
Bulk RNA sequencing (RNAseq) analysis reveals significant differences in genetic profiles and pathways of SCZ oligodendrocytes. **(A)** Clustering of bulk RNAseq data with dimensionality reduction by principal component analysis (PCA). Ctrl iOLs are labeled by purple circle, and SCZ iOLs are labeled by magenta triangle **(B)** Volcano plot depicts the genes identified from differential expression analysis in SCZ iOLs versus Ctrl iOLs. Red and blue points label the genes with significantly increased or decreased expression respectively in SCZ iOLs, the dashed horizontal line corresponds to a cut-off significance threshold of *p*-value < 0.05, and the dashed vertical lines indicate the fold change thresholds (log_2_FoldChange >= 1.5 or log_2_FoldChange <= −1.5). Scatter plots of canonical pathway (CP) analysis show **(C)** the top 10 upregulated reactome pathways and **(D)** the top 10 downregulated reactome pathways. The number of differentially expressed genes in the pathway is represented by the circle size, and the colors of circle indicate the normalized enrichment score (NES). Data are based on biological independent hiPSC lines from 8 patients with SCZ and 7 Ctrl.

To investigate transcriptomic signatures in parallel to the morphological assessment, we performed bulk RNAseq of Day +10 iOLs. Dimensional reduction with PCA showed that the general genetic features of iOLs from SCZ patients and Ctrl did not differ (Fig. 6A). However, differential gene expression analysis identified 93 upregulated and downregulated genes in iOLs from SCZ patients compared to Ctrl with *p*-values < 0.05 (Fig. 6B, Table S4). Notably, only the upregulated gene *POTEM* remained significant after FDR correction, while 23 genes were significant after *p*-value adjustment applying the simpleM approach [51], that accounts for the elevated correlation within the transcriptomic data to estimate the effective number of tests. To gain more insights for the differentially expressed genes (DEGs), we conducted a Pubmed search with the keyword of the individual gene and oligodendrocyte and schizophrenia, respectively. Only few DEGs such as *APOE* were previously linked to oligodendrocyte (9 out of 93) and/or schizophrenia (18 out of 93), while the majority of DEGs have not yet been investigated for their roles in oligodendrocyte and schizophrenia (Table S5).

Given that individual genes may not fully reflect the effects of biological pathways alterations, we conducted a gene set enrichment analysis (GSEA) across all expressed genes to uncover the underlying biological information [52] of transcriptomic alterations in SCZ. For the GSEA, we applied the standard *q*-value threshold of 0.25. Most upregulated pathways in iOLs from SCZ patients are relevant to G protein-coupled receptors (GPCR) pathways, cell signaling or immune response (Fig. 6C, Table S6). While the top 10 downregulated pathways in iOLs from SCZ patients are all involved in DNA replication or cell cycles (Fig. 6D, Table S6). These findings reveal differences in expression profiles between iOLs from SCZ patients and Ctrl, and dysregulated pathways relevant to signaling mechanisms, DNA replication and cell cycle regulation are noteworthy.

## Discussion

In this study, we found evidence for the genetic impact of SCZ on mature iOLs and revealed morphological alterations in iOLs from patients with SCZ. First, unsupervised clustering of transcriptomes of hiPSC-derived iOPCs/iOLs and human postmortem OPCs/OLs revealed a high similarity underlining the validity of studying human oligodendroglial cells in vitro with hiPSC technology. Second, by performing gene set enrichment analyses, we found an enrichment of SCZ risk in maturating human iOLs, supporting previous findings with new technology [21, 22, 54]. This result implies that the polygenic architecture of schizophrenia could have a significant impact on OL genes, particularly those expressed on advanced stages of OL maturation. Of note, our performed gene enrichment analysis was based on hiPSC-derived cells, and that were analyzed with scRNAseq, but not single-nucleus RNA sequencing (snRNAseq) or perinuclear scRNAseq data from postmortem brains or mouse-derived samples [19, 20]. Postmortem and whole mouse brain tissues can only be processed for snRNAseq but not scRNAseq, because it is technically impossible to dissociate intact cells from postmortem brain tissues [55]. Consequently, our scRNAseq readouts of late-stage iOPCs/iOLs also contained broad cytoplasmic transcripts that could explain, at least in part, the differences between the results of our gene enrichment analysis and previous findings [19, 20]. Moreover, transcriptomic profiles from aged OLs obtained via postmortem sampling may not reflect the critical OL cell stage implicated in SCZ. Of note, in line with previous findings indicating an oligodendroglial contribution in BPD [56, 57], iOL2 genes were also slightly associated with BPD, that is genetically correlated with SCZ [58]. This result raises questions regarding the specificity of the oligodendroglial component in the genetic architecture of SCZ, suggesting a potential common mechanism for both disorders related to this cell type.

Notably, DTI imaging within our translational cohort confirmed the reduced whole brain FA as an indicator for white matter disturbances in line with a previous multicenter study of 4322 individuals that linked global FA alteration in SCZ to altered oligodendroglial microstructure [4]. Subsequently, we selected representative SCZ patient showing pronounced white matter disturbances and unaffected Ctrl samples with hiPSCs available from our translational cohort [33] for subsequent hiPSC modelling. With patient-derived iOPC/iOL monoculture from the imaging-based patient representatives, we aimed to investigate whether there are cell-autonomous disturbances of the oligodendroglial lineage in SCZ.

Interestingly, our automated morphological image analyses revealed altered morphological phenotypes in MBP^+^ mature iOLs from SCZ patients, including longer branch length and increased junction numbers, but no measurable alterations were found in O4^+^ late-stage iOPCs/premyelinating iOLs from SCZ patients. Importantly, only the mixed-effects ANOVA, that provides the sensitivity to detect group-level effects despite variability among individual lines, revealed the significance of morphological differences. In comparison, simpler methods such as t-tests on aggregated group-level averages did not yield statistically significant results. This discrepancy underscores the value of mixed-effects models in capturing subtle effects that might be masked by group-level variability. Thus, our findings suggest that joint polygenic mechanisms in SCZ drive cellular alterations in the oligodendroglial lineage in monocultures despite the genetic variability of the hiPSC donors [36].

However, it also highlights a limitation: mixed-effects models are more sensitive to sample size, and their robustness can be limited in studies with small cohorts. Although in line with a previous recommendation for the design of hiPSC cohort composition [37], future studies should aim to increase the sample size to address this limitation by encompassing both a greater number of patient-derived cell lines and technical replicates. The scalability of our developed iOL differentiation protocol will allow to do this in the future [24]. Moreover, complementing morphological analyses with functional assays, such as studying myelination in more complex systems (e.g., nanofibers or neuroglial co-culturing systems in 2D, 3D, or chimeric models), could help confirm the biological relevance of the observed morphological alterations and provide a more comprehensive understanding of SCZ-associated changes in oligodendroglial function [59–61]. A further technical limitation is the presence of non-oligodendroglial cells within our directed differentiations approach. Our previous technical study revealed around 8.8% neuronal cells present in SON-induced cells [24], suggesting the potential existence of cell types other than oligodendrocyte lineages in our cultures that could potentially influence the morphological and transcriptomic assessments of cell-autonomous effects of oligodendrocyte lineages. Furthermore, although the data were derived from biologically independent hiPSC lines from different patients with schizophrenia and the significant effects in MBP^+^ iOLs reflected consistent group-level trends across these lines, it is important to note that the results were obtained from a single parallel oligodendroglial differentiation across the cohort.

Most previous results with postmortem samples showing OLs disturbances and white matter deficits in SCZ revealed the dysregulation of OL-relevant genes, declined number of OLs, and decreased volume of white matter, suggesting dysregulated oligodendrocytes and impaired myelination in the brains of adult and aged SCZ patients [4, 13, 14, 62]. A previous study also generated hiPSC-derived OLs from SCZ patients with a chemical differentiation protocol and found significantly decreased number of O4^+^ cells after 85 days in culture [63]. Importantly, the in vitro model we used applies directed oligodendroglial differentiation with induced overexpression of lineage-determining transcription factors and may reflect different differentiation stages compared to the previous study [63]. Moreover, iPSC-based analysis investigates much earlier developmental stages compared to postmortem samples of SCZ. The increased branch length and junction numbers in SCZ iOLs likely represent an increased maturation speed that might misalign with trophic signals from developing axons, which might indirectly impact on the survival rate of OLs, known to be dependent on neuronal activity [64]. To what extent our findings of disturbed morphology of iOLs are in line with a previous study, which also revealed a prematuration phenotype in the migration of SCZ glial progenitor cells into the cortex using a humanized glial chimeric mouse model [65], should be addressed in future studies.

To investigate the transcriptomic underpinnings of the morphological alteration, we performed an RNAseq investigation. It should be noted that the DEG analysis was limited by the relatively small cohort size, and thus the identified individual genes may not fully reflect the effects of biological alteration associated with SCZ. Notably, only one gene, *POTEM*, reached significance by conservative FDR-adjusted *p*-value correction. Although not an established standard in RNAseq analysis, multiple testing correction using the simpleM approach accounting for the effective number of independent tests [51], that is becoming increasingly adopted in the field of biological psychiatry [66, 67], identified more than 20 DEGs, suggesting that overly conservative approaches may overlook disease signals in genetically complex and heterogeneous diseases such as SCZ.

To gain further insights into the biological dysfunctions contributed by the joint effects of certain gene sets in SCZ, a GSEA [52] was performed assessing all the expressed genes for pathway analysis. Thereby, several altered signaling pathways of the oligodendroglial lineage in SCZ were identified, in line with the prematuration hypothesis. The top downregulated pathways were related to replication and cell cycle regulation. Thus, the attenuation of these pathways could support a hypothesis of a prematuration phenotype in SCZ that would also fit with the abnormal morphological iOL phenotypes in SCZ as we have observed. The differential expression analysis identified several dysregulated genes in SCZ iOLs, while only a few among them were investigated in the context of oligodendrocyte and/or schizophrenia before. Among the dysregulated genes, *APOE* encodes the protein APOE which regulates lipid metabolism and myelination and plays an important role in synaptic plasticity and cognition [68]. *APOE* is not only involved in Alzheimer’s disease [68] but also associated with SCZ, as studies have demonstrated its upregulation in SCZ [69].

Taken our observations together, we hypothesize that the irregular maturation of oligodendroglial cells in SCZ may impede the formation of normal neuronal networks, leading to a secondary loss of OLs and disrupted connections among brain regions and thus ultimately contributing to impaired cognitive functions. Nonetheless, we did not investigate the process of myelination and assess functions of neuronal networks, which could be addressed with electrophysiological tests of microcircuits in OLs co-cultured with neurons or in 3D cellular systems such as myelinating neurospheres or organoids [70, 71]. Another prematuration phenotype at the structural level has been described in a recent study in major depressive disorder that revealed morphological changes in a hiPSC-derived neuronal model with increased neurite length in selective serotonin reuptake inhibitor (SSRI)-non-responder subgroup [72]. Although this study is confined to a neuronal phenotype, it may relate to our study showing that a defined genetic background can cause a prematuration phenotype in a cellular model.

In summary, we validated iPSC-modelling as a robust tool for studying human oligodendroglial cells and demonstrated the importance of OLs in SCZ by revealing the enrichment of oligodendroglial genes in SCZ GWAS and morphological and transcriptomic dysregulation of SCZ-derived iOLs. These results suggest a failed maturation phenotype in oligodendroglial cells of patients with SCZ, which could contribute to the widely observed white matter pathology in SCZ. However, further investigations in escalated cohorts and functional assessments are needed to gain a more mechanical understanding of the pathophysiology of the widely observed oligodendroglial disturbances in SCZ.

## Supplementary information

Supplementary information

Table S1. Lists of gene sets for MAGMA gene enrichment analysis

Table S2. Test statistics of DTI and ICC imaging analysis

Table S4. Lists of DESeq differentially expressed genes

Table S5. Number of articles of DESeq genes from Pubmed search

Table S6. Lists of GSEA Reactome pathways

## Data Availability

Any patient-related materials that can be shared will be released via material transfer agreement and data processing agreement, conditional on proper ethics approval from the requesting institution. A subset of iPSC lines is subject to sharing constraints due to limited donor consent.

## Code availability

The developed, automated imaging pipeline for the morphological quantification of the oligodendroglial cells has been made publicly available (last update on 30^th^ of April 2024): https://github.com/skoghoern/cell-coloc-and-morphology.
