# Supplementary material for: iPSC-modelling reveals genetic associations and morphological alterations of oligodendrocytes in schizophrenia
Source: Transl Psychiatry. 2025 Aug 16;15:287. doi: 10.1038/s41398-025-03509-x (PMC12357907; doi:10.1038/s41398-025-03509-x)
Supplement: Supplementary file 1 — Supplementary information [file 41398_2025_3509_MOESM1_ESM.pdf]

Supplementary information

iPSC-modelling reveals genetic associations and morphological alterations of oligodendrocytes in schizophrenia

Chang, Waldeck *et al.*

Corresponding authors:

Moritz J. Rossner, Department of Psychiatry and Psychotherapy, LMU University Hospital, LMU Munich, Nußbaumstraße 7, 80336 Munich, Germany, Mail: moritz.rossner@med.uni-muenchen.de

Florian J. Raabe, Max Planck Institute of Psychiatry, Kraepelinstr. 2-10, 80804 Munich, Germany, Mail: florian_raabe@psych.mpg.de

Supplementary tables

Table S1. Lists of gene sets for MAGMA gene enrichment analysis
Generated gene sets from scRNAseq profiling by Raabe et al. [24], characterizing different cells and differentiation stages within the established directed oligodendroglial differentiation protocol. The *p*-value of the association of each gene with SCZ risk by MAGMA analysis is annotated. See separate excel file.

Table S2. Test statistics of DTI and ICC imaging analysis
See separate excel file.

Table S3. Cohort information with unique iPSC ID on hPSCreg

Sample ID	Unique iPSC ID - hPSCreg.eu	Sex	Age
Ctrl1	PSYLMUi002-A	Male	27
Ctrl2	PSYLMUi008-A	Male	35
Ctrl3	PSYLMUi009-A	Male	27
Ctrl4	PSYLMUi011-A	Male	33
Ctrl5	PSYLMUi003-A	Male	21
Ctrl6	PSYLMUi004-A	Female	34
Ctrl7	PSYLMUi006-A	Male	53
SCZ1	PSYLMUi035-A	Male	34
SCZ2	PSYLMUi018-A	Male	45
SCZ3	PSYLMUi023-A	Male	50
SCZ4	PSYLMUi026-A	Female	29
SCZ5	PSYLMUi027-A	Male	55
SCZ6	PSYLMUi029-A	Male	37
SCZ7	PSYLMUi030-A	Male	20
SCZ8	PSYLMUi031-A	Male	54

Table S4. Lists of DESeq differentially expressed genes
See separate excel file.

Table S5. Number of articles of DESeq genes from Pubmed search
See separate excel file.

Table S6. Lists of GSEA Reactome pathways
See separate excel file.

Supplementary figures

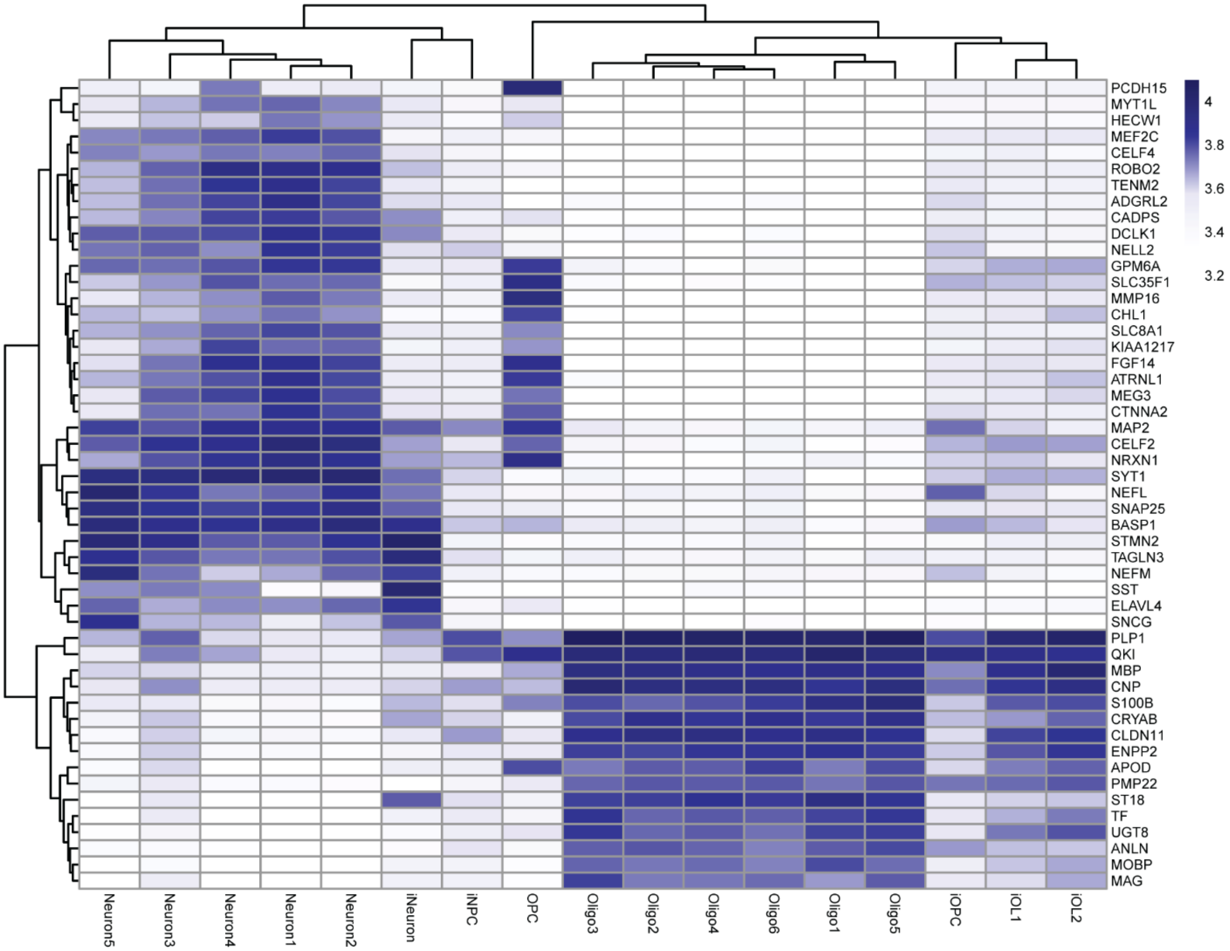

Figure S1. hiPSC-derived iOPCs and iOLs cluster with human postmortem OPCs and OLs. Heatmap of the top 50 gene hits from the clustering analysis of hiPSC-derived cells [24] with human postmortem brain cells [27]. Unsupervised hierarchical clustering was performed. *Abbreviations: OPCs, human postmortem oligodendrocyte precursor cells; Oligo1-6, human postmortem oligodendrocytes; Neuron1-5, human postmortem neurons; iOPC, hiPSC-derived OPC; iOL1-2, hiPSC-derived oligodendrocytes; iNPC, hiPSC-derived neural precursor cell; iNeuron, hiPSC-derived neurons.*

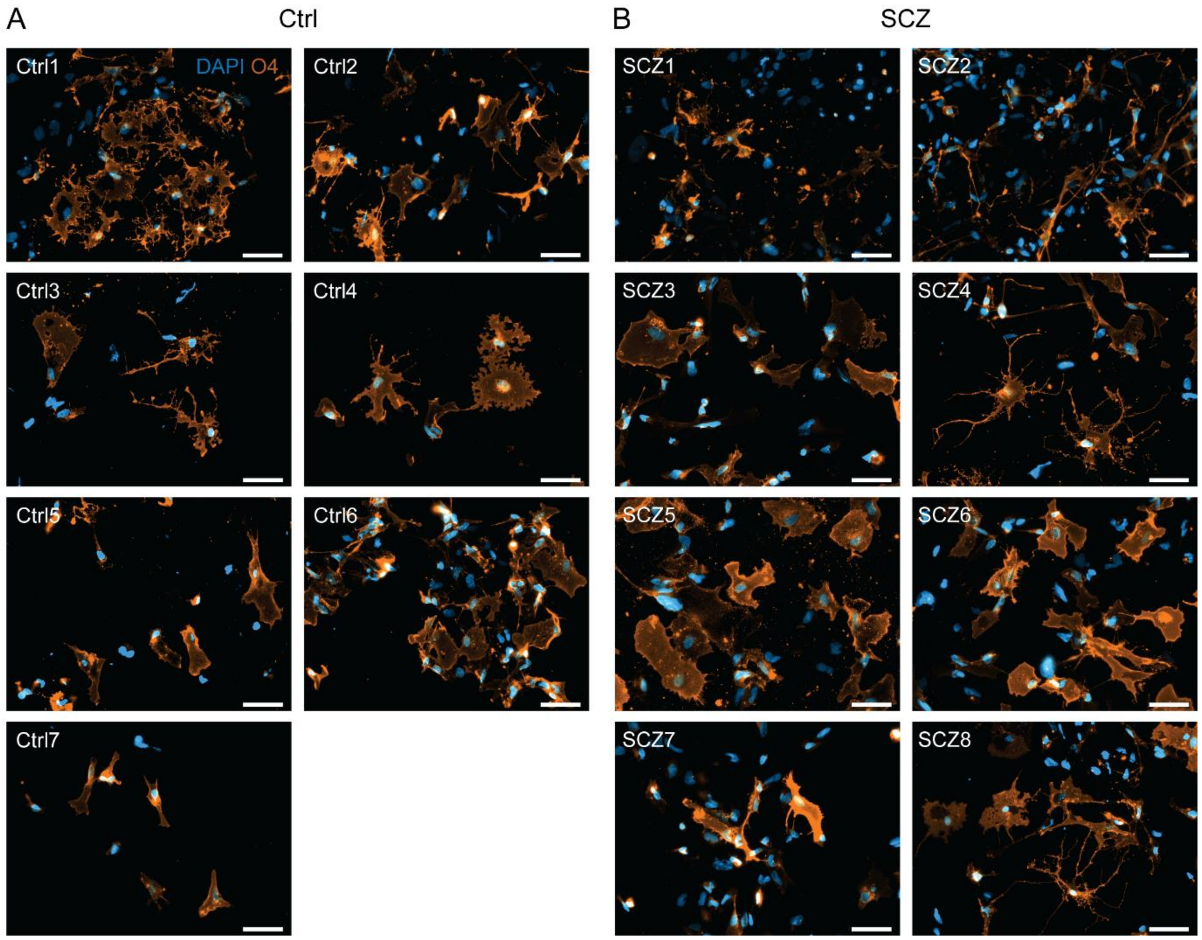

Figure S2. Oligodendroglial differentiation allows the generation of hiPSC-derived O4$^+$ premature iOPCs.
(A) Representative images of control cell lines. **(B)** Representative images of SCZ cell lines. Scale bar indicates 50 µm.

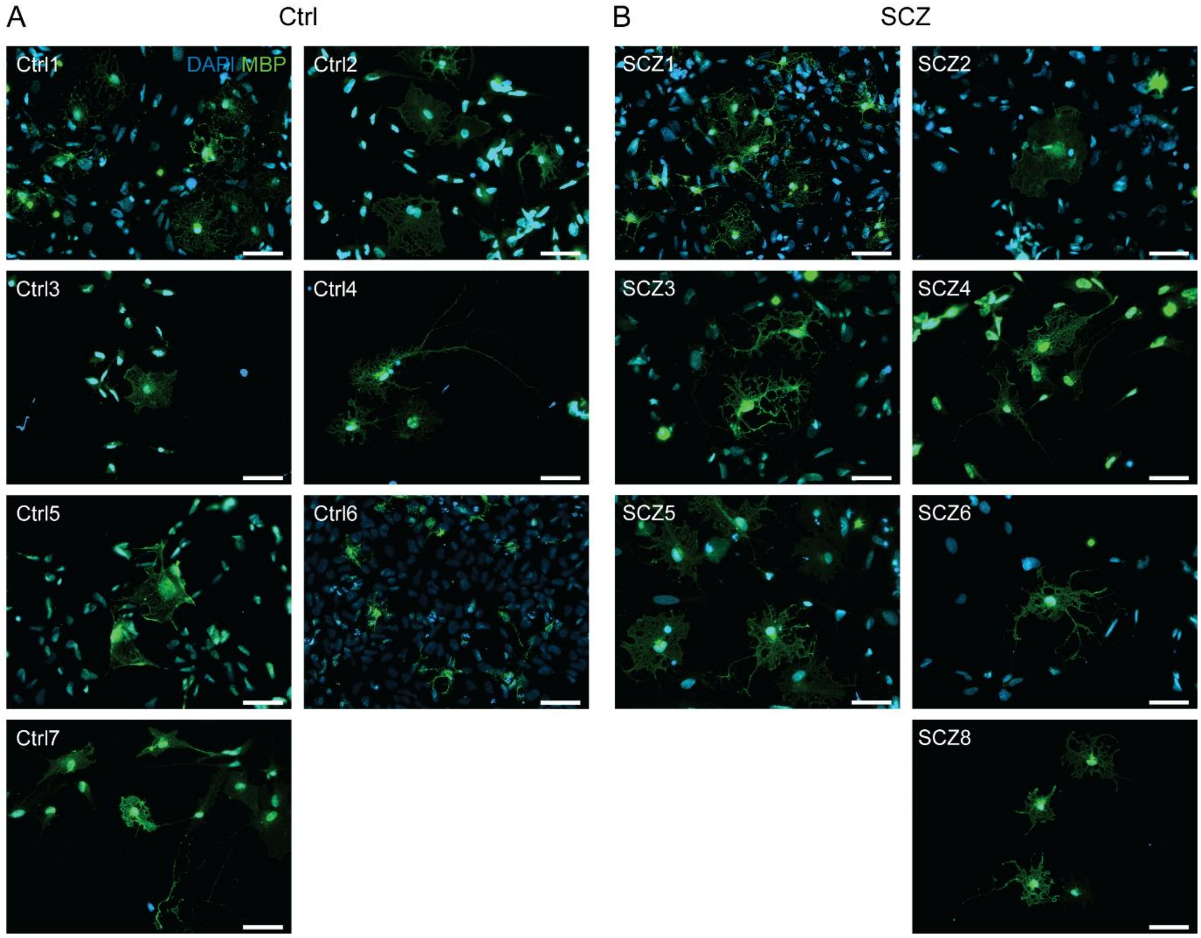

Figure S3. Oligodendroglial differentiation allows the generation of hiPSC-derived MBP⁺ iOLs.
(A) Representative images of control cell lines. **(B)** Representative images of SCZ cell lines. Scale bar indicates 50 µm.

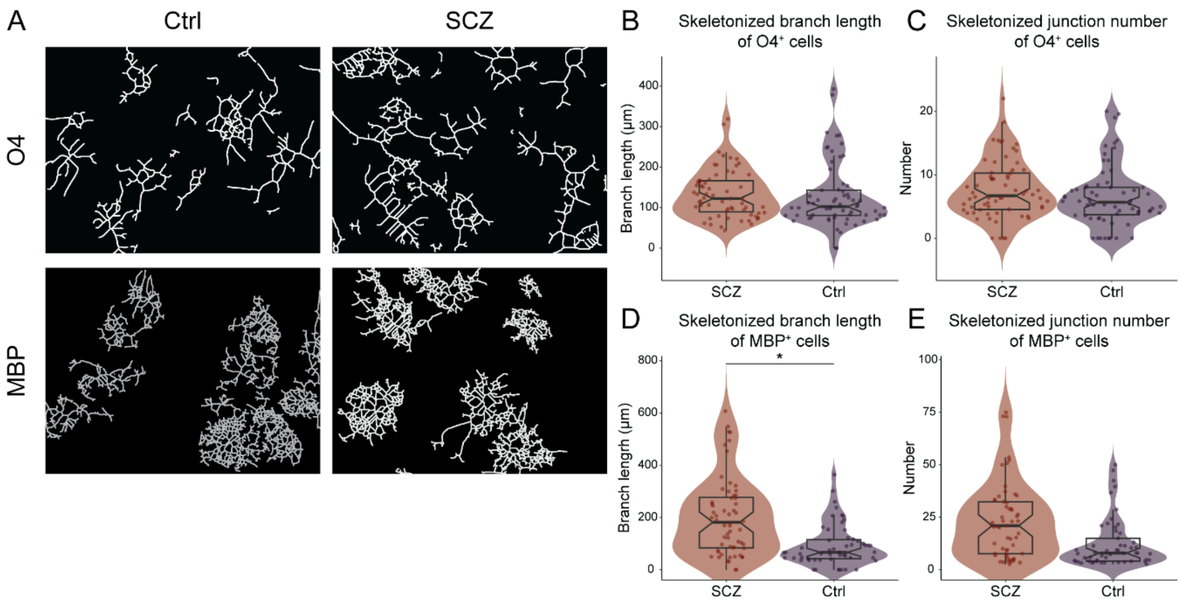

Figure S4. Validation of the morphological findings with an alternative morphological quantification analysis method.

(A) (Top) Representative image of O4+ iOPCs from Ctrl and SCZ patients of **Figure 4A** after applying *skeletonize* as morphological quantification method. (Bottom) Representative image of MBP+ iOLs from Ctrl and SCZ patients of **Figure 5A** after applying *skeletonize* as morphological quantification method. Boxplots illustrate quantification of **(B)** average branch length of O4+ iOPCs and **(C)** average junction number of O4+ iOPCs (Ctrl, N = 7, n = 10 fields of view / cell line, vs. SCZ, N = 8, n = 8-10 fields of view / cell line), and quantification of **(D)** average branch length of MBP+ iOLs (Ctrl, n = 65 fields of view, N = 7, n = 8-10 fields of view / cell line, vs. SCZ, N = 7, n = 8-10 fields of view / cell line) and **(E)** average junction number of MBP+ iOLs (Ctrl, N = 7, n = 8-10 fields of view / cell line, vs. SCZ, N = 7, n = 8-10 fields of view / cell line,). Data are based on biological independent hiPSC lines from 8 patients with SCZ and 7 Ctrl. Scale bar indicates 50 μm, Data = Mean ± SD. Mixed-effects ANOVA with group identity (SCZ-Ctrl) as the between-group factor: *, $p < 0.05$.

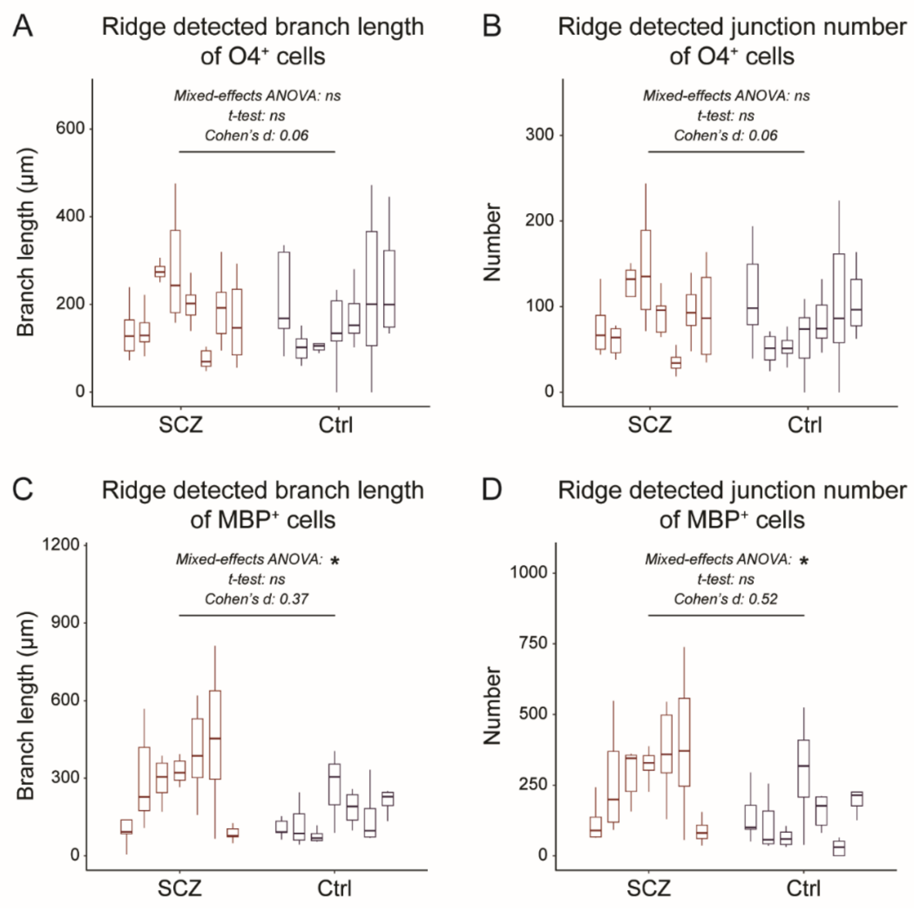

Figure S5. Morphological assessment across individual cell lines.
Boxplots illustrate **(A)** the branch length and **(B)** the junction number from each cell line of $O4^+$ iOPCs. A t-test and Cohen's d were used to compare the average values of each cell line on group level (Ctrl, N = 7, vs. SCZ, N = 8) and a mixed-effects ANOVA was performed to account for repeated measures across all images taken (Ctrl, n = 10 fields of view / cell line, vs. SCZ, n = 8-10 fields of view / cell line). Data are based on biological independent hiPSC lines from 8 patients with SCZ and 7 Ctrl. Boxplots illustrate **(C)** the branch length and **(D)** the junction number from each cell line of MBP^+ iOLs. A t-test and Cohen's d were used to compare the average values of each cell line (Ctrl, N = 7, vs. SCZ, N = 7) and a mixed-effects ANOVA was performed to account for repeated measures across all images taken (Ctrl, n = 8-10 fields of view / cell line, vs. SCZ, n = 8-10 fields of view / cell line). Data are based on biological independent hiPSC lines from 7 patients with SCZ and 7 Ctrl. ns, not significant; *, $p < 0.05$.
